# An analytical model of college students’ self-assessed satisfaction with the effectiveness of online learning: a structural equation model integrating LICE and S-O-R models

**DOI:** 10.3389/fpsyg.2023.1248729

**Published:** 2024-04-08

**Authors:** Xin Dai, Rong rong Wang, Xue feng Huang, Xiao xue Wang, Ya ting Huang, Yingying Li, Yuqing Wu, Chong yuan Guan, Regina Queen Kazembe, Yuanyuan Zhang, Bo Gao

**Affiliations:** ^1^School of Public Health, Dalian Medical University, Dalian, China; ^2^School of International Education, Dalian Medical University, Dalian, China; ^3^Department of Psychology, Dalian Medical University, Dalian, China

**Keywords:** S-O-R model, LICE model, online learning, learning motivation, online learning effect satisfaction

## Abstract

**Background:**

Nowadays, e-learning significantly affects college students’ academic life. This study aims to examine the factors that influence college students’ satisfaction with online learning outcomes.

**Method:**

The study population consisted of undergraduate students from Dalian Medical University, with a total of 715 college students participating in the study. Out of these participants, 602 valid questionnaires were obtained. Demographic data was analyzed using SPSS.22, and the data was cleaned and prepared for testing the research hypotheses. The proposed research framework was examined using structural equation modeling (SEM) through Smart-PLS 3.0.

**Results:**

The results of the study showed that student satisfaction with learning outcomes was positively correlated with several factors: quality of teacher instruction (*β* = 0.100, *p* < 0.0001), quality of e-learning platforms (*β* = 0.059, *p* < 0.0001), individual learner factors such as learning motivation (*β* = 0.112, *p* < 0.001), and e-learning environment (*β* = 0.469, *p* < 0.001). Additionally, self-learning efficacy (*β* = 0.081, *p* < 0.0001), learning strategies (*β* = 0.031, *p* < 0.001), and learning motivation (*β* = 0.039, *p* < 0.001) were found to have mediating effects.

**Conclusion:**

Understanding the satisfaction of college students with the effect of e-learning holds great significance in coping with teaching methods in unexpected situations. It enables adjustments to teaching strategies, improvements to learning platforms, and mobilization of students’ motivation. Thus, it serves as a valuable reference in addressing unexpected teaching scenarios.

## 1. Introduction

### 1.1. Research background

E-learning has become a necessary mode of learning in the current education system. The rapid development of online courses has been fueled by the increasing demand for both new and existing courses. Studies have shown that about 66% of institutions in the U.S. reported a rise in demand for new online courses (Allen and Seaman, 2010). Similarly, approximately 73% of educational institutions observed an increase in demand for existing online courses (Alqurashi, 2016). The COVID-19 pandemic, along with other global health crises, has further highlighted the limitations of traditional education, such as spatial constraints and limitations in timeliness. This has exposed the contradiction between existing intelligent educational technology and the current needs of higher education, thereby accelerating the progress of online courses (Pokhrel and Chhetri, 2021). To meet the demands of the digital era, higher education institutions have integrated modern information technology with teaching activities, constructing new teaching platforms that utilize e-learning (Ma, 2017). As a result, e-learning has become an indispensable part of college students’ daily study lives.

The pandemic has provided an opportunity for the rapid development of innovative and alternative education systems and assessment strategies, paving the way for the introduction and promotion of digital learning (Xie et al., 2020). Consequently, much research has been focused on optimizing e-learning, which includes improving the learning environment, reforming teaching methods, upgrading educational resources, and fostering students’ learning expectations (Wang et al., 2021). However, despite these advancements, various factors continue to impact college students’ experience in e-learning, leading to persistent challenges. Some of these challenges include the absence of an effective online learning atmosphere (Zhang, 2021), low learning atmosphere, inadequate interactivity in virtual classrooms, and limited teacher-student interaction (Dai and Lin, 2020). Furthermore, the use of a single teaching method, the lack of effective assessment methods, insufficient online course resources (Zhou et al., 2020), and inadequate learning platforms and technical support contribute to the ongoing issues with e-learning (Bao, 2020).

According to studies, continuous exposure to shortcomings in e-learning can increase the pressure on learners’ perceived ease of use and usefulness of new technologies. This, in turn, affects users’ satisfaction with e-learning and may lead to negative attitudes or rejection of e-learning (Kauffman, 2015; Chen et al., 2020; Alqahtani et al., 2022). Additionally, a survey by Maria S Abbasi found that satisfaction with e-learning is higher in developed countries than in developing countries. Most participants agreed that e-learning provides satisfactory access to knowledge (Abbasi et al., 2020). Zhang et al. (2021) demonstrated a significant correlation between college students’ self-efficacy, motivation to learn, and student satisfaction. Consequently, it is crucial to explore and enhance the factors that enable learners to persist in e-learning in order to increase satisfaction. This will be particularly important in regional education systems with an equal level of economic development, as it will facilitate a successful educational process (Elshami et al., 2021).

### 1.2. Online education hotspot cluster analysis

**Figure 1:**
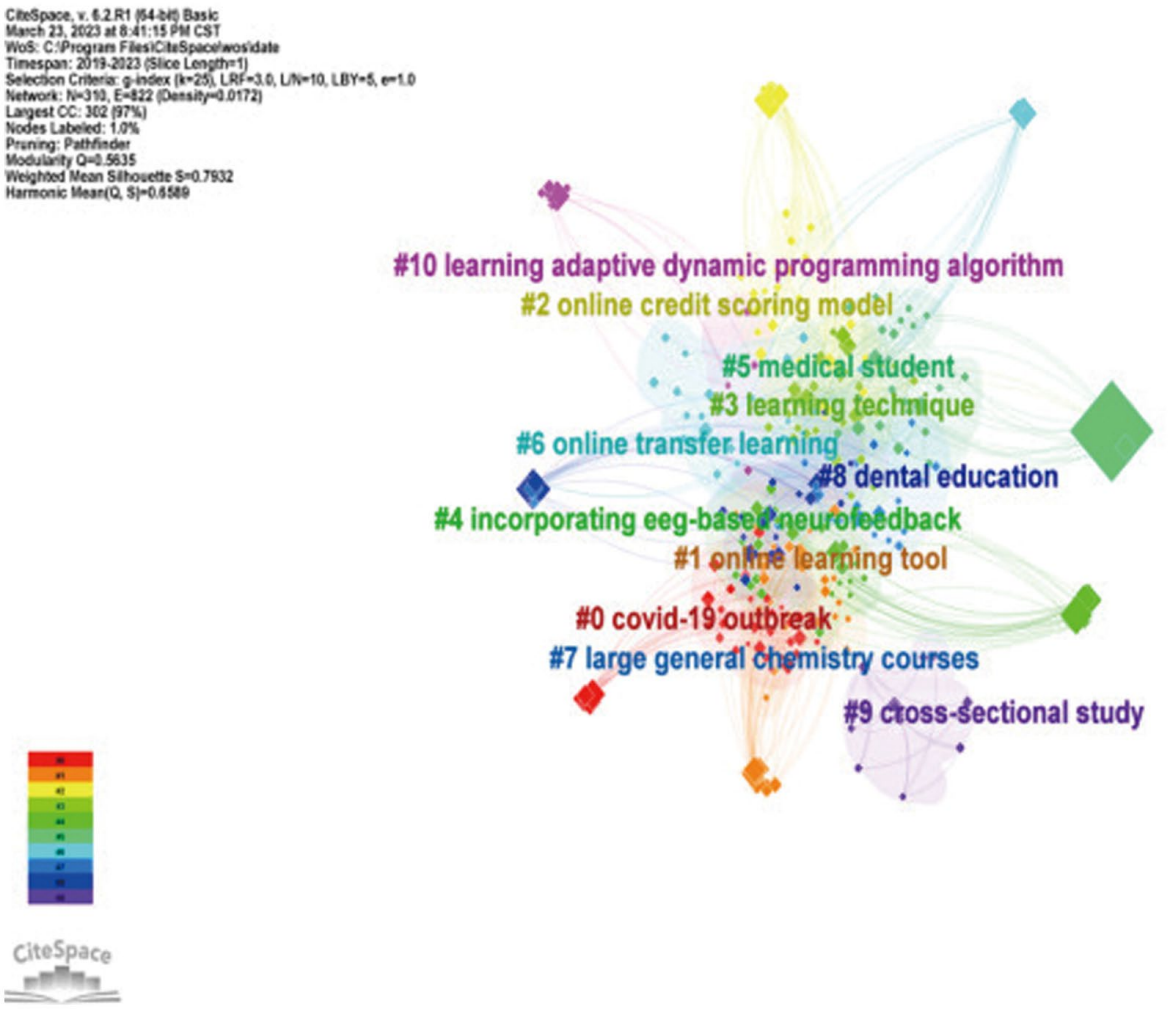
Visual mapping of keyword clustering.

In general, the number of articles related to online learning research has increased significantly due to the influence of COVID-19 from January 2020 to January 2023, as evidenced by records from Web of Science and PUB MED database. As shown in Figure 1, the research hotspots within this field, identified through keyword co-occurrence and clustering analysis, mainly revolve around the learning effect of e-learning, teaching strategies, independent learning, and student performance. However, despite the growing importance of online learning, little attention has been given to the factors that impact student satisfaction with their learning outcomes. Consequently, it is crucial to address this research gap. Notably, e-learning research has gained attention in various regions, such as North America (e.g., Canada), Europe (e.g., United Kingdom), Asia (e.g., China, India, Australia), and Africa. Building upon this background, the current study primarily aims to investigate the factors influencing satisfaction with e-learning outcomes comprehensively.

### 1.3. Satisfaction with the effectiveness of e-learning

Traditionally, a student’s subjective evaluation of their educational experience is known as student satisfaction (Elliot and Shin, 2002). Satisfaction is measured by students after performing a learning activity (Li, 2018). Student satisfaction reflects learning outcomes between students and teachers (Yunusa and Umar, 2021). Nelson sees learning satisfaction as a combination of good perceptions and positive attitudes (Nelson, 2022). This is because students can combine the two to meet their individual needs in the learning process. Students’ satisfaction with online learning requires advanced pedagogical methods and technological know-how to capture students’ attention and teaching (Baber, 2020). Learning satisfaction is a feeling and attitude toward the learning process; this feeling and attitude is formed by the pleasure students feel when the learning activity or process meets their physical and mental needs (Li, 2018). Gopal et al.’s (2021) study found that the perceived quality of the instructor, course design, instructor feedback, and student expectations were the most important predictors of student satisfaction. Satisfaction with the effectiveness of online learning among college students is an important variable to explore, which is important to facilitate the understanding of the current state of learning among college students.

### 1.4. Stimulus-Organism-Response (S-O-R) theoretical framework

The S-O-R model, developed by Mehrabian and Russell (1974), suggests that when a person is exposed to an external stimulus, certain cognitive or affective states will be evoked and an individual response will be triggered. This model involves three theoretical components: stimulus, organism, and response. It assumes that stimuli (S) in the external environment lead to changes in people’s internal organisms (O), which in turn affects their behavioral responses (R). The model has been widely used in various domains, including retail purchasing behavior, social media engagement, online user behavior, and educational instruction, to explain how external characteristics (i.e., stimulus variables) influence human internal thoughts (i.e., organism variables) and behavioral responses (i.e., response variables) (Zhao et al., 2020). In this theoretical framework, stimuli appear in different forms, such as environmental factors and interpersonal relationships (Animesh et al., 2011). As the global COVID-19 pandemic has led to a shift from offline to online classrooms, students are now forced to experiment with multimedia tools for learning (Khan et al., 2017). Yan Zhan and colleagues found that the S-O-R model is suitable for studying the context of online learning user behavior (Zhai et al., 2020). Their study concluded that the stimuli perceived during e-learning can be considered as stimuli from the external environment that stimulate the learner’s behavior, including motivation, learning strategies, and self-directed learning ability, and ultimately affect the learner’s learning outcomes.

### 1.5. LICE model of online learning influencing factors

**Figure 2:**
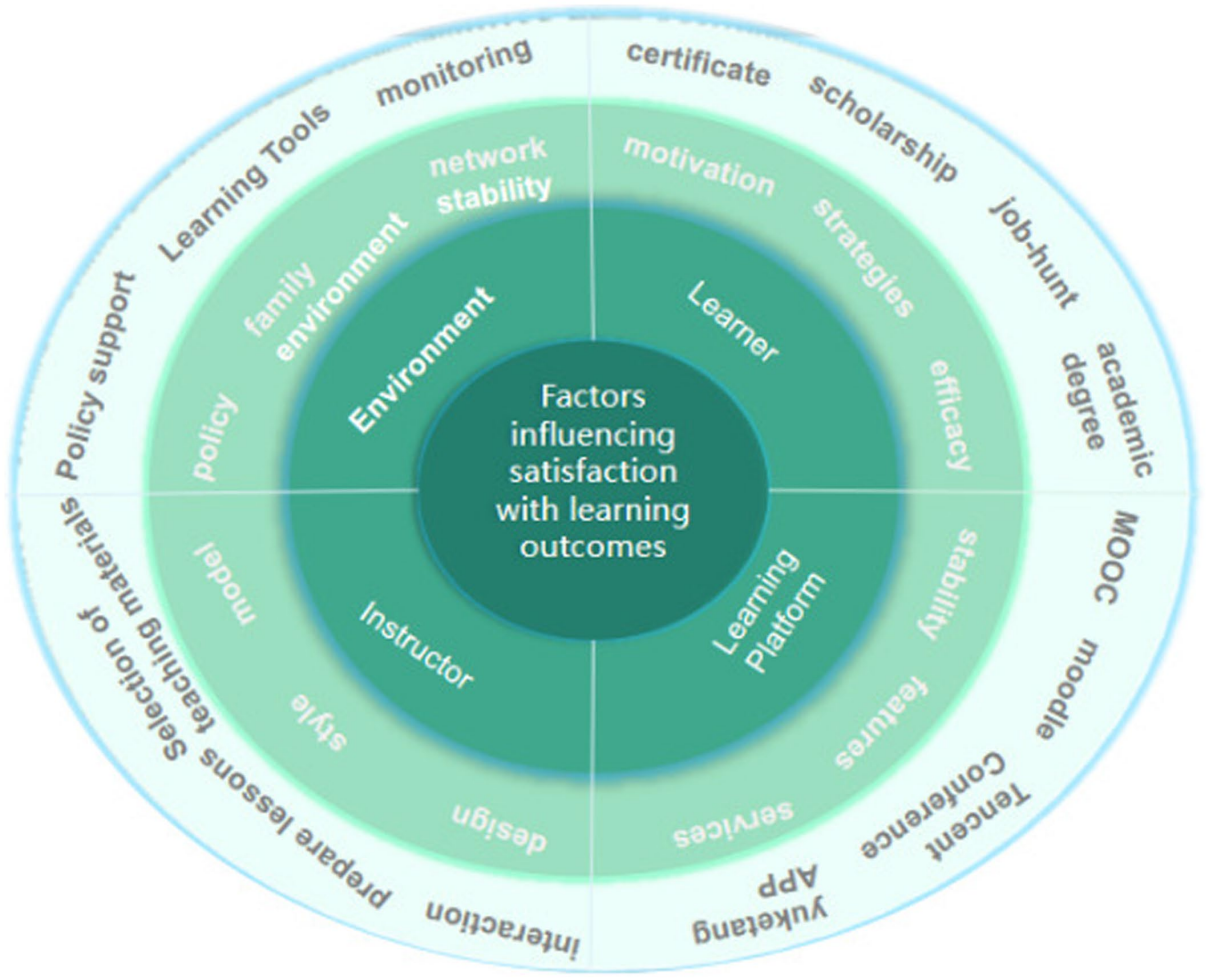
LIPE model.

The LICE model of factors affecting e-learning, proposed by Chinese scholar Jiahua Zhang, categorizes these factors into four aspects: Learner, Instructor, Curriculum, and Environment (referred to as the LICE model by Zhang and Jianping, 2009). Based on this model, along with related research both domestically and internationally, this study proposes a model that describes the factors affecting network learning, as depicted in Figure 2. The model divides the influencing factors into four aspects: Learner, Instructor, Learning platform, and Learning environment, leading to the abbreviation LIPE model. In this model, the learner factor serves as the key internal influencing factor of network learning, while the Instructor is an important factor. Additionally, the learning environment serves as a guarantee factor, and the learning platform acts as an auxiliary factor. These three factors together constitute the external influencing factors of network learning. It is the collaboration between the internal and external influencing factors that ultimately impacts the effectiveness of e-learning.

This study combines the research hotspots in the field of online education and integrates the structure of the mature LICE online education evaluation model. It introduces the LIPE model for online learning satisfaction assessment and focuses on exploring the comprehensive factors affecting students’ satisfaction with the effect of online learning. With the help of the Stimulus-Organism-Response (S-O-R) theoretical framework, it provides realistic strategies for adjusting the teaching strategy, improving the learning platform, and mobilizing the students’ motivation for online learning. The results of the study promote the long-lasting and sustainable development of online education and provide value for coping with the sudden change of the teaching mode in unexpected situations.

## 2. Method

### 2.1. Research model and hypothesis

**Figure 3:**
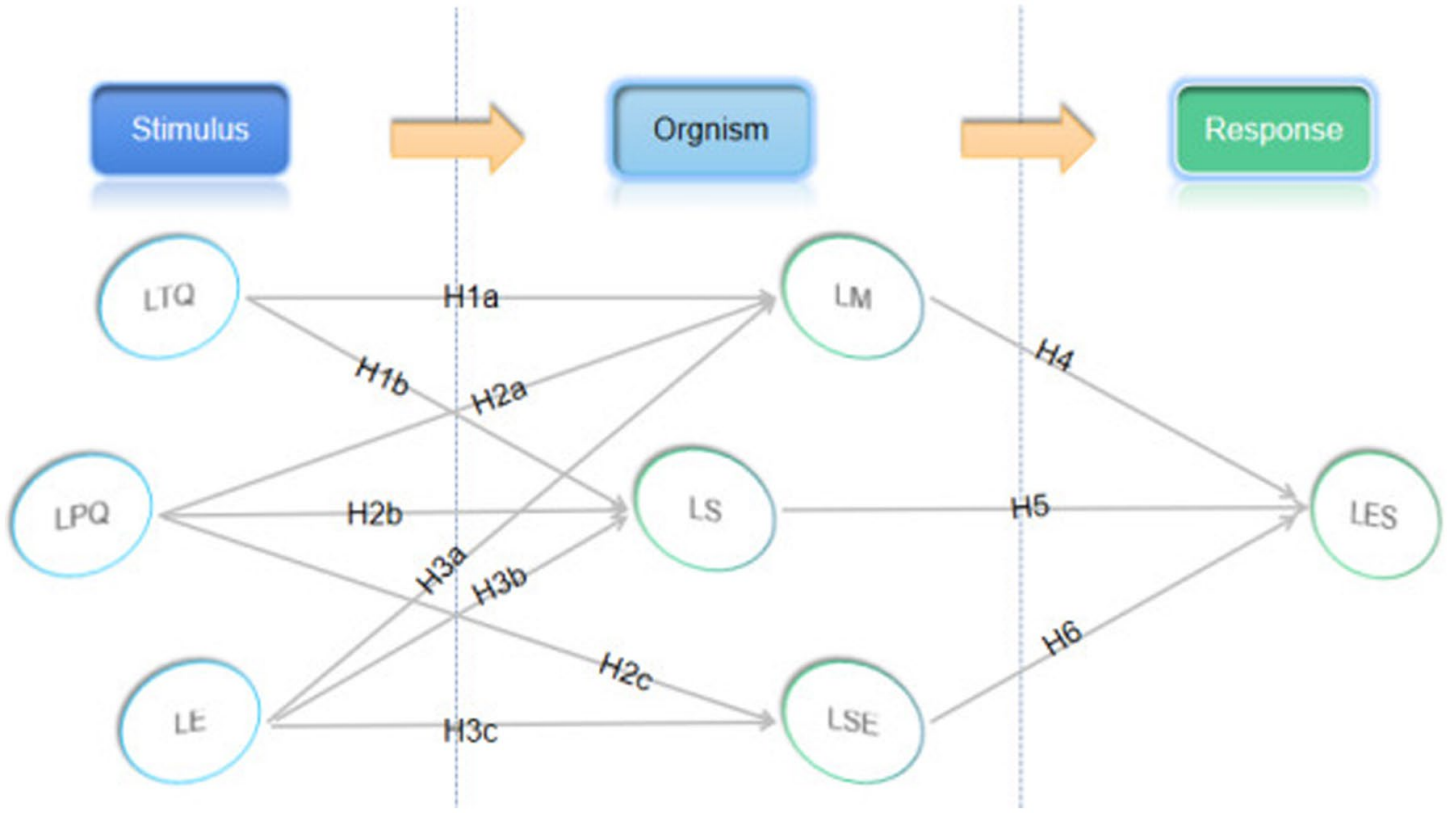
Research framework and hypothesis.

Based on the S-O-R model, this study aimed to investigate the effects of learning platform quality, instructor teaching quality, and learning environment as stimulus variables on learners’ satisfaction with e-learning outcomes when they are engaged in e-learning. To further identify the biological variables in this study, we integrated the LICE model with the S-O-R model, according to which four variables (i.e., instructional teacher factors, learning platform factors, learning environment, and learner factors) were considered as biological and environmental variables in the S-O-R framework. Based on this, this study developed a research hypothesis model combining the LICE model and the S-O-R model, as shown in Figure 3. Based on the research model, several hypotheses were developed.

#### 2.1.1. Relationship between the quality of teaching by lecturers and individual learner factors

In the process of E-learning, the teaching quality of educators plays a crucial role in influencing the effectiveness of online education. Zhang et al. (2020) argue that online teaching not only helps to reduce cross-transmission in traditional classrooms but also measures online student satisfaction, thus ensuring the quality of online teaching during a pandemic. The effect of E-learning on students is also influenced by various factors such as the teaching style of educators, their interaction with students, and the courseware and teaching materials used. Manou et al. (2021) propose that an increased frequency of instructor-student interactions in e-learning courses leads to better course quality and improved student learning outcomes. Additionally, Horntvedt et al. (2018) found that the use of interactive instructional strategies further enhances students’ knowledge and skills. Based on these findings, it can be concluded that there is a positive relationship between the teaching quality of educators and both learning motivation and learning strategies in the process of E-learning. Therefore, the following hypotheses are proposed:

*H1a*: There is a positive relationship between the teaching quality of educators and learning motivation in the process of E-learning.

*H1b*: There is a positive relationship between the teaching quality of educators and learning strategies.

#### 2.1.2. The relationship between the quality of e-learning platform and the personal factors of learners

In China, online education began in the 1990s. The e-learning platform, known as a comprehensive teaching service support system, offers features such as teaching tutorials, online self-learning, and online teacher-student communication. It provides an environment for distance learning. The widespread adoption of online learning was accelerated by the impact of the COVID-19 pandemic on offline teaching in 2019. As a result, various online learning platforms, such as Learning Pass, MOOC, Tencent Meeting, Moodle, and others, have continued to improve their development. Research studies have demonstrated that students have no difficulty using these platforms. Moreover, the effectiveness of the learning platforms, coupled with positive communication between instructors and students, has led to students expressing their desire for online learning to continue even after the pandemic (Adeyeye et al., 2022). This is further supported by the findings of Ogura et al. (2018) who discovered that medical students’ learning outcomes can be effectively enhanced through the use of e-learning platforms. Similarly, Yakin and Linden (2021) found that students taught via e-learning platforms performed better on laboratory exams compared to those who were not.

*H2a*: There is a positive relationship between the quality of e-learning platforms and learning motivation.

*H2b*: A positive correlation between the quality of e-learning platforms and Personal learning strategies.

*H2c*: A positive relationship between the quality of e-learning platforms and self-efficacy.

#### 2.1.3. The relationship between online learning environments and personal factors of learners

Bandura’s Ternary Interactive Determinism (TID) considers that the environment, individual cognition, and behavior are independent, interdependent, and mutually influential, and emphasizes the influence of the individual’s environment on his or her behavior (Bandura, 1977). Findings suggest that the learning environment is an important determinant of students’ cognitive and affective outcomes (Eom et al., 2006). Many studies confirm the relationship between students’ perceptions of the learning environment and their affective learning outcomes (Cho and Cho, 2014). Junqi Zhu and other scholars believe that the effect of college students’ online learning is mainly influenced by potential variables such as online learning behavior, online learning cognition, and online learning environment (Zhu et al., 2022). Therefore, the environmental factors of e-learning are included in the influences that affect the effectiveness of e-learning.

*H3a*: There is a positive relationship between the online learning environment and learning motivation.

*H3b*: There is a positive relationship between the online learning environment and Personal learning strategies.

*H3c*: Online learning environment has a positive relationship with self-efficacy.

#### 2.1.4. The relationship between learning strategies, learning motivation, and satisfaction with the effect of online learning

E-learning includes online lectures, audio-visual aids (AVA), PowerPoint slides, and recorded video lessons (Baber, 2020). Students’ feelings, attitudes, and hopes about the quality of the learning environment as an expression of satisfaction with learning (Wu et al., 2010), and were widely used indicators of students’ attitudes toward the online learning process (Graham and Scarborough, 2001). Motivation for learning is one of the important variables in emotion-based research and refers to the internal (intrinsic) and external (extrinsic) forces that empower students to learn effectively (Keller, 2007). Learning strategies are the process of acquiring, organizing, or transforming information (Alexander et al., 1998). Studies have shown a significant positive correlation between students’ use of these strategies and learning satisfaction (Choi, 2016). Muis et al. (2015) found that by effectively using learning strategies to removes barriers to learning, reduces negative emotions, and allow students to enjoy learning. Chang et al. (2017) showed that interest in utilizing e-learning resources as well as their performance and motivation influenced their desire to embrace e-learning. Voils et al. (2019) found positive associations between the use of instructor-created supplemental activities and classroom test scores, self-regulated learning behaviors, and overall course performance.

*H4*: There is a positive correlation between learners' motivation and satisfaction with learning outcomes.

*H5*: There is a positive correlation between learners' learning strategies and satisfaction with learning outcomes.

#### 2.1.5. The relationship between self-efficacy and satisfaction with learning outcomes

Self-efficacy theory was proposed by Bandura, an American psychologist, who suggested that the interaction between environment, behavior, and person affects individual behavior, emphasizing the mediating and regulating role of self-factors on behavior (Bandura, 1977). Self-efficacy indicates the degree to which an individual possesses the ability to manage a task or stress, and the concept is also related to motivation levels, actions, and psychological states (Zimmerman and Kulikowich, 2016). In their study of the relationship between motivation and academic performance among medical students, Wu Hongbin and other scholars found that both intrinsic and extrinsic motivation predicted self-efficacy (Wu et al., 2020).

*H6*: A positive relationship between self-efficacy and satisfaction with the effectiveness of online learning.

#### 2.1.6. The mediating role of learning motivation, learning strategies, and self-efficacy

Bandura’s Ternary Interactive Determinism (TID) posits that the environment, individual cognition, and behavior mutually influence each other, indicating that the individual’s environment has a significant impact on their behavior (Bandura, 1977). Consistent with this perspective, Mohd Satar et al. (2021) discovered that e-learning satisfaction and key outcomes were mediated by learning stress and motivation among students. Furthermore, Doménech-Betoret et al. (2017) found that self-efficacy plays a crucial role in influencing academic performance. Based on these findings, we propose the following hypothesis:

*H7*: Learning environment influences satisfaction with the effectiveness of online learning through self-efficacy.

*H8*: Quality of e-learning platforms influences satisfaction with the effectiveness of online learning through learning strategies.

*H9*: The teaching quality of educators affects satisfaction with the effectiveness of online learning through learning motivation.

### 2.2. Participants

A total of 715 people, comprising of undergraduate students in grades 1 to 5 on campus at a medical university in Dalian, were surveyed between October 2021 and December 2022. Among the 602 recovered valid questionnaires, the effective response rate was 84.2%. The demographic information of the college students interviewed is shown in Table 1. Out of the respondents, 312 were male, representing 51.8%, while 290 were female, representing 48.2%. The majority of the respondents, 89.9%, belonged to the ethnic group, while the remaining 10.1% were from other ethnic minorities. In terms of academic year, 47.5% were freshmen, 14.8% were sophomores, 6.8% were juniors, 12.8% were seniors, and the remaining 18.1% were seniors and above. Medical students constituted the largest group, accounting for 45.6% of the respondents, followed by management students at 12.5%, art students at 6.6%, humanities and social sciences students at 5.3%, law students at 5.0%, science and engineering students at 8.1%, and literature students at 9.6%.

**Table 1:** Demographic information of study participants (N-602).

Sample characteristics	Category	Sample		Sample characteristics	Category	Sample	
		Number	Proportion/%			Number	Proportion/%
Gender	Male	312	51.8%	Ethnic	Chinese	540	89.9%
	Female	290	48.2%		Minority	61	10.1%
Grade	Freshman	286	47.5%	Specialized	Medicine	318	52.8%
					Management	75	12.5%
	Sophomore	89	14.8%		Art	40	6.6%
					Humanities and social sciences	32	5.3%
	Junior	41	6.8%				
	Senior	71	12.8%		Legal studies	30	5.0%
	Fifth year	109	18.1%		Science and engineering	48	8.1%
					literary	58	9.6%

### 2.3. Research design

#### 2.3.1. Sampling and questionnaire distribution

To investigate college students’ satisfaction with learning outcomes and their influencing factors, this study commenced with face-to-face interviews and the distribution of questionnaires using stratified random and purposive sampling. The samples of this study primarily consisted of undergraduate students in the first to fifth year of a medical university in Dalian. A total of 715 questionnaires were distributed to universities in schools, and 602 valid questionnaires were recovered, resulting in an effective response rate of 84.2%. The purpose of this study was to test the aforementioned hypotheses and explore the relationship between latent and observed variables.

#### 2.3.2. Inclusion and exclusion criteria

The following data inclusion and exclusion criteria were set to ensure data validity and minimize errors:

1. The questionnaires were collected for undergraduate students in the first to fifth year of a medical university in Dalian.
2. Studying the impact of e-learning on satisfaction with learning outcomes, so traditional ways of learning are ruled out.
3. Data from college students who had engaged in online learning were included, and students who had never had online learning experience were excluded.

### 2.4. Research tool

The questionnaire was divided into two parts. The first part included demographic information such as gender, major, grade level, etc. The second part consists of four dimensions, including four parts: learning environment, teacher teaching, learning platform, and individual learner, which are used to measure students’ satisfaction with the effect of e-learning. All four dimensions were utilized in the Rilicet Response Scale. The questionnaire used in this study was designed according to the needs of the study, therefore the reliability and validity of the questionnaire were tested.

#### 2.4.1. Questionnaire reliability test

In this study, the internal consistency method was used to test the reliability of the variables and to analyze the reliability of the questionnaire items. The alpha reliability coefficient of the measured variables was calculated to be 0.993, which is an acceptable value with α > 0.7 indicating high reliability of the questionnaire items.

#### 2.4.2. Questionnaire validity test

The study used factor analysis to analyze the validity of the questionnaire scale construct using KMO and Bartlett’s test of sphericity. The results showed that the KMO value of the scale was 0.967 (>0.8), which is close to 1, indicating that the sample size meets the requirements and the data are suitable for factor analysis, while Bartlett’s test of the sphericity significance level of *p* = 0.000 < 0.01 indicates that the original variables are meaningful and the scale data are suitable for factor analysis.

In summary, the questionnaire has good reliability and validity.

### 2.5. Data collection and data analysis

Data were collected from October 2021–December 2022 and the questionnaires were all electronic and distributed through the WJX platform. The data were then entered and managed using Excel and analyzed using SPSS.22 for descriptive statistical analysis and correlation analysis. Finally, the hypothesized model was validated with the help of Smart PLS and using SEM analysis.

## 3. Results

### 3.1. Multivariate analysis

#### 3.1.1. Measurement model evaluation

Structural equation modeling (SEM) allows the use of multiple observations for each latent variable to explain measurement error in both the dependent and independent variables (Levine et al., 2005). First, a correlated validated factor measurement model with 8 variables was constructed using Smart PLS 3.0. Secondly, SEM analysis was performed to measure the path coefficients as well as the fit coefficients of the models. To test the reliability of the scale this study was validated in terms of measuring the item aspects and latent variables, respectively. The measurement item aspect was assessed using factors and coefficients. As shown in Table 2, all projects have loads above the 2010 Hair et al. (2010) recommendation of 0.50 and were significant at the 0.001 level. Latent variable validation was measured using the combined reliability and Cronbach’s α values, and both the combined reliability and ɑ values were taken to be greater than the suggested threshold of 0.7, and the AVE values for all items were greater than 0.5.

**Table 2:** Validity of model convergence.

Construct	Item	Factor loading	Cronbach’s α	CR	AVE
E-learning platform quality (LPQ)	LPQ1	0.887	0.725	0.879	0.784
	LPQ2	0.884			
E-learning teaching quality (LTQ)	LTQ1	0.894	0.841	0.904	0.759
	LTQ2	0.852			
	LTQ3	0.867			
E-learning environment (LE)	LE1	0.852	0.769	0.867	0.685
	LE2	0.826			
	LE3	0.804			
E-learning motivation (LM)	LM1	0.822	0.811	0.876	0.639
	LM2	0.807			
	LM3	0.808			
	LM4	0.759			
E-learning strategies (LS)	LS1	0.851	0.815	0.890	0.729
	LS2	0.845			
	LS3	0.866			
E-learning self-efficacy (LSE)	LSE1	0.802	0.773	0.869	0.688
	LSE2	0.845			
	LSE3	0.840			
E-learning effects satisfaction (LES)	LES1	0.881	0.870	0.911	0.720
	LES2	0.859			
	LES3	0.826			
	LES4	0.827			

#### 3.1.2. Discriminant validity

**Figure 4:**
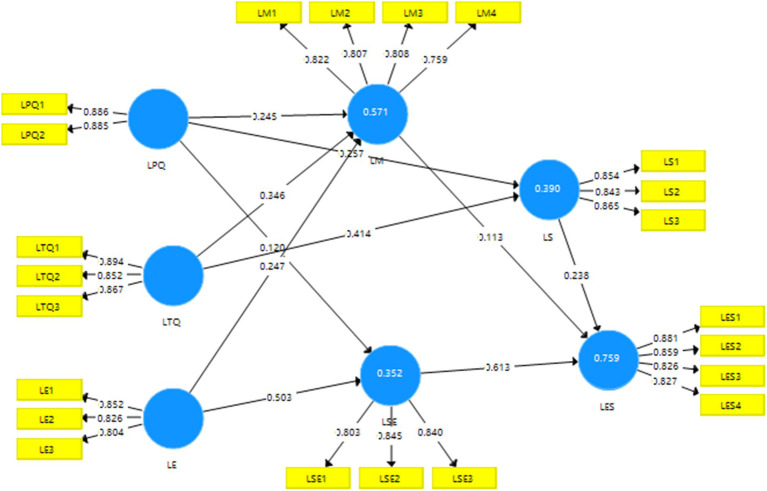
PLS-SEM.

Validity is a measure of whether a comprehensive evaluation system accurately reflects the purpose and requirements of the evaluation. It refers to the degree of accuracy in measuring the specific characteristics to be measured. Higher validity indicates a better ability of the measurement tool to label the intended characteristic, and vice versa. Discriminant validity (DV) can be used to quantify concepts that are not conceptually related to each other. Discriminant validation aims to provide evidence of discrimination based on differences between all components (Campbell and Fiske, 1959). The distinctiveness of validity can be measured using the square root of the average variance extracted (AVE) compared to the magnitude of the correlation coefficient value. In Table 3, correlation coefficients between the constructs that are less than the square root of the AVE indicate good discriminant validity. The value of convergent validity, measured by AVE, in Table 3 and Figure 4, ranged from 0.603–0.723, which is greater than 0.5. Therefore, it can be concluded that the model demonstrates good validity and reliability.

**Table 3:** Convergence validity of the model.

Construct	AVE	LE	LES	LM	LPQ	LS	LSE	LTQ
LE	0.685	**0.827**						
LES	0.720	0.593	**0.849**					
LM	0.639	0.670	0.657	**0.799**				
LPQ	0.784	0.701	0.533	0.666	**0.886**			
LS	0.729	0.619	0.722	0.707	0.554	**0.854**		
LSE	0.688	0.587	0.839	0.614	0.472	0.659	**0.829**	
LTQ	0.759	0.727	0.540	0.701	0.717	0.598	0.485	**0.871**

Meaning of bolded values square root of AVE.

### 3.2. Structural equation model

#### 3.2.1. Model fitting

Structural modeling is a theoretical framework for evaluating internal path models using structural equations (Skrondal and Rabe-Hesketh, 2004), assuming a series of regression equations to estimate the structural model coefficients of the inter-construct relationships. Model fit is measured using the Standardized Root Mean Square Residual (SRMR), a standardized residual index used to assess model fit, chi-square, and the Normal Fit Index (NFI) (Brown, 2006; Chen, 2007). The SRMR value compares the observed covariance with the predicted matrix, and the result is better when it is less than or equal to 0.08. The predicted SRMR value is 0.053, which is a good model fit; the NFI is 0.808; and the chi-square value is 1587.353, as shown in Table 4.

**Table 4:** Model fit summary.

Estimated model	
SRMR	0.053
d_uls	0.721
d_G	0.445
chi-square	1587.353
NFI	0.808

#### 3.2.2. Model results

**Figure 5:**
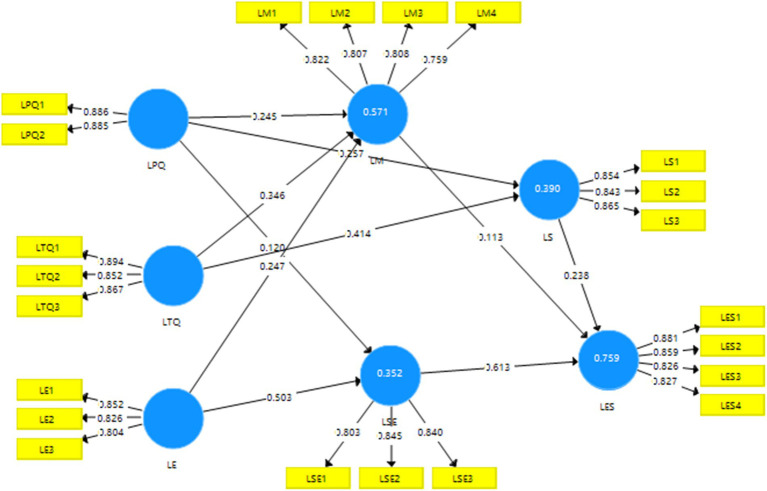
Structural equation model results.

When evaluating structural relationships, it is important to check for covariance to ensure that it does not bias the regression results; the variance inflation factor (VIF) is commonly used to assess the covariance of exogenous structures (Hair et al., 2019). In the study, the VIF values for each construct were below the threshold value of 5.0 and very close to 3. This indicates that the problem of covariance between constructs does not exist and is in line with Hair et al.’s VIF cutoff of 5.0, which is in line with Hair et al.’s recommendation that the VIF scores should be close to 3 and that a lower value is needed. Table 5 and Figure 5 show that almost all exogenous variables have positive and statistically significant relationships with the hypothesized endogenous variables.

**Table 5:** Results of structural model evaluation of direct relationships.

Hypothesis	Relationship	Path coefficients	VIF	S.D	*P*	Hypothetical test results
H1	H1a:LTQ → LM	0.346	2.587	0.044	0.000	Supported
	H1b: LTQ → LS	0.225	2.587	0.051	0.000	Supported
H2	H2a: LPQ → LM	0.245	2.396	0.047	0.000	Supported
	H2b: LPQ → LS	0.132	2.396	0.046	0.005	Supported
	H2c: LPQ → LSE	0.120	1.966	0.054	0.027	Supported
H3	H3a: LE → LM	0.247	2.471	0.047	0.000	Supported
	H3b: LE → LS	0.341	2.471	0.053	0.000	Supported
	H3c: LE → LSE	0.503	1.966	0.054	0.000	Supported
H4	LM → LES	0.113	2.167	0.033	0.001	Supported
H5	LS → LES	0.238	2.387	0.035	0.000	Supported
H6	LSE → LES	0.612	1.917	0.033	0.000	Supported

Based on the results presented in Figure 5 and Table 5, the quality of teacher’s instruction exhibits a positive correlation with both personal motivation to learn and personal learning strategies (*β* = 0.346, *p* < 0.001; *β* = 0.255, *p* < 0.001), thus confirming the validity of hypotheses H1a and H1b. Similarly, the quality of e-learning platforms demonstrates a positive correlation with individual motivation to learn, supporting hypothesis H2a (*β* = 0.245, *p* < 0.001). Additionally, the quality of e-learning platforms is positively associated with individual learning strategies and the efficacy of individual self-directed learning, reinforcing hypotheses H2b and H2c (*β* = 0.132, *p* < 0.005; *β* = 0.120, *p* < 0.005). Furthermore, a positive relationship emerges between the e-learning environment and learning motivation, learning strategies, and personal efficacy in self-directed learning, thereby confirming hypotheses H3a, H3b, and H3c (*β* = 0.247, *p* < 0.001; *β* = 0.341, *p* < 0.001; and *β* = 0.503, *p* < 0.001). Moreover, a positive correlation is observed between learning motivation and learning effectiveness, verifying hypothesis H4 (*β* = 0.113, *p* < 0.001). Similarly, a positive correlation exists between personal learning strategies and learning effectiveness, confirming hypothesis H5 (*β* = 0.238, *p* < 0.001). Finally, a positive correlation is found between personal independent learning efficacy and learning effectiveness, supporting hypothesis H6 (*β* = 0.612, *p* < 0.001).

#### 3.2.3. Intermediary role

To examine the mediating role of learning strategies, motivation, and self-learning effectiveness, Baron and Kenny’s (1986) research process was employed in this study. The independent variables included learning environment, quality of learning platform, and quality of teachers’ teaching, while the mediating variables were learning strategies, motivation, and self-learning efficacy. The dependent variable was satisfaction with learning outcomes. Data analysis was conducted using Smart PLS 3.0, with the mediation effect assessed through Bootstrapping at a 95% confidence interval. The results in Table 6 show a positive correlation between the learning environment and personal independent learning efficacy (*β* = 0.503, *p* < 0.001), as well as between personal independent learning efficacy and satisfaction with learning outcomes (*β* = 0.612, *p* < 0.001). Furthermore, the quality of the learning platform had a positive effect on online learning strategies (*β* = 0.132, *p* < 0.001), which in turn positively influenced satisfaction with learning outcomes (*β* = 0.238, *p* < 0.001). Similarly, teacher teaching quality had a positive effect on online learning motivation (*β* = 0.346, *p* < 0.001), and online learning motivation had a positive impact on satisfaction with learning outcomes (*β* = 0.113, *p* < 0.001). The assessment of specific indirect effects in Table 7 revealed that self-learning efficacy mediated the relationship between the learning environment and satisfaction with learning outcomes (*β* = 0.089, *p* < 0.001), supporting research hypothesis H7. Additionally, learning strategies mediated the relationship between the quality of learning platforms and satisfaction with learning outcomes (*β* = 0.031, *p* < 0.001), confirming research hypothesis H8. Moreover, motivation played a significant mediating role in the relationship between teachers’ teaching quality and satisfaction with learning outcomes (*β* = 0.039, *p* < 0.001), establishing research hypothesis H9.

**Table 6:** Total indirect effects of the standard model.

Hypothesis	SIE	*β*	M	S.D	*T*	*P* values	Confidence intervals	
							2.5%	97.5%
H7	LE- > LSE	0.503	0.504	0.048	7.030	0.000	0.395	0.611
	LSE- > LES	0.612	0.611	0.034	18.170	0.000	0.540	0.674
H8	LPQ- > LS	0.132	0.132	0.046	2.892	0.004	0.051	0.221
	LS - > LES	0.238	0.237	0.036	6.657	0.000	0.163	0.308
H9	LTQ- > LM	0.346	0.343	0.048	7.186	0.000	0.248	0.427
	LM - > LES	0.113	0.115	0.030	3.722	0.000	0.063	0.181

**Table 7:** Results of the assessment of specific indirect effects.

Hypothesis	SIE	*β*	M	S.D	*T*	*P* values	Confidence intervals	
							2.5%	97.5%
H7	LE- > LSE- > LES	0.089	0.307	0.034	9.006	0.000	0.053	0.111
H8	LPQ - > LS - > LES	0.031	0.031	0.012	2.521	0.012	0.011	0.060
H9	LTQ - > LM - > LES	0.039	0.040	0.013	3.112	0.002	0.018	0.064

## 4. Discussion

With the continued resurgence or crossover of different risk pandemics, many schools are rapidly adopting online delivery models, enhancing the planning of e-learning technology and systems platforms so that students can continue to receive their education (Wang et al., 2022). This not only changes the traditional mode of learning for students but also makes the continued implementation of online learning an important issue for the sustainability of education in the post-epidemic era. Since the end of 2021, the associated risks and regular vaccinations have had a significant impact on the social and economic livelihoods of individuals in the post-COVID-19 era (Cheng et al., 2021), including in the area of online education. Therefore, exploring the factors that influence students to engage in online learning has become an important research topic. The results of this study showed that the quality of teachers’ teaching, the learning environment, the quality of the learning platform, and personal factors (learning motivation, learning strategies, and self-efficacy) have a great impact on the satisfaction of e-learning outcomes. Taking the students of a medical university in Dalian as a sample, the S-O-R model was used to conduct a substantial study to explore the influence of the above factors on the satisfaction of the e-learning effect. This study fills the gap of the influence factors of college students’ online learning affect satisfaction in the context of online learning (Zhang et al., 2021).

### 4.1. Factors affecting satisfaction with e-learning outcomes

#### 4.1.1. Personal factors influencing the effectiveness of online learning among college students

**Figure 6:**
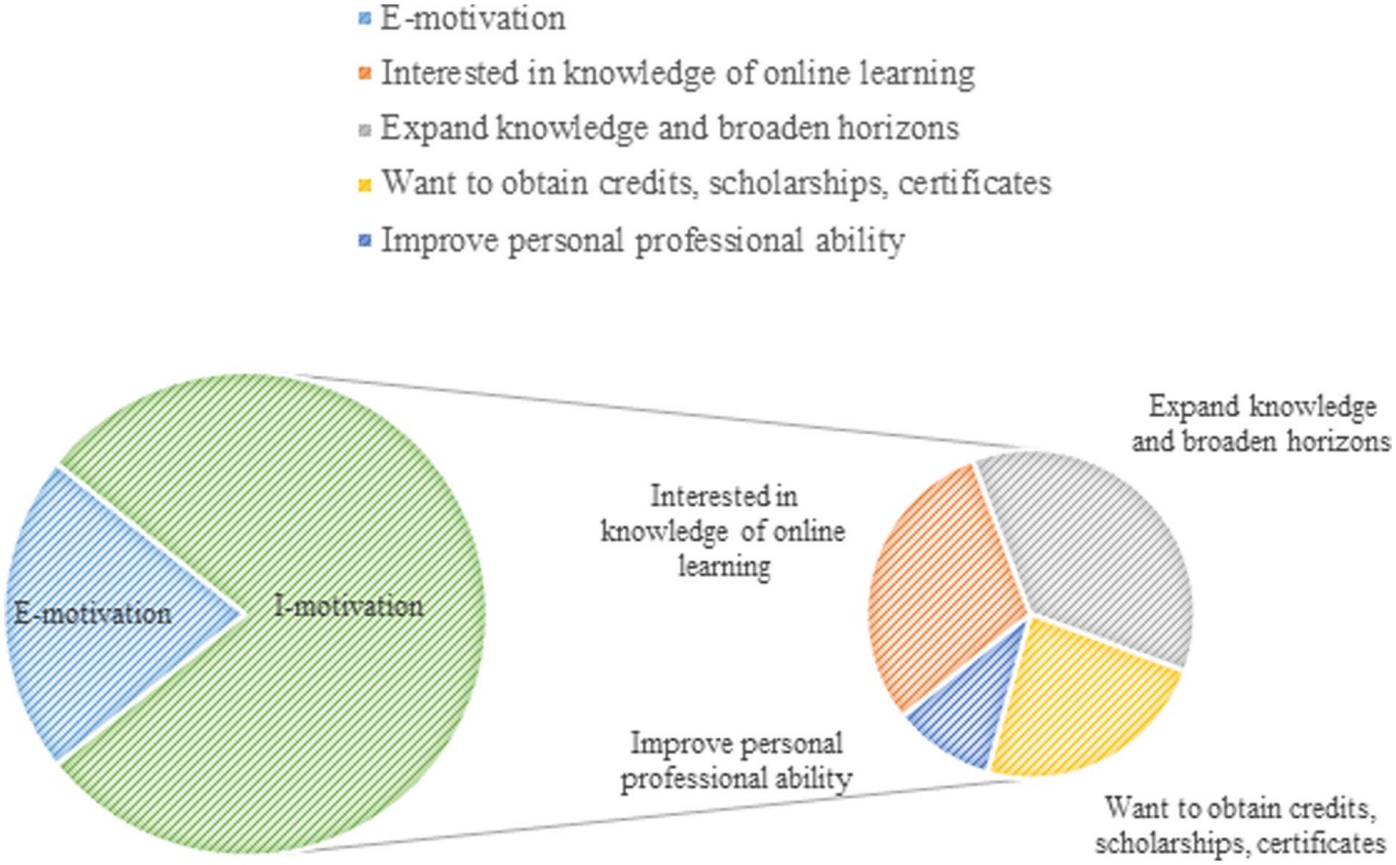
Comparison of internal and external motivations of college students in online learning.

The results of this study indicate that learners’ personal motivation, learning strategies, and self-efficacy when engaging in e-learning have an impact on learners’ satisfaction with learning outcomes. In a study on the impact of college students’ adaptability to blended learning in English, Shuhan Yang and other scholars also found that self-efficacy and motivation had a significant positive impact on the adaptability of non-English majors to the blended learning mode of English courses (Yang and Pu, 2022). Therefore, individual learner factors are crucial in influencing the satisfaction of e-learning outcomes and are very well guided in enhancing and promoting the user experience of the blended learning services offered. In addition, it was found that many students actively used e-learning platforms to acquire knowledge to further their studies and obtain certificates, etc. Interestingly, such groups tended to report higher e-learning effectiveness and satisfaction than those who passively accepted e-learning platforms (Figure 6). According to the results of the study we found that there is a significant positive correlation between motivation, learning strategies, self-efficacy, and e-learning effectiveness, which is consistent with the findings of Wlodkowski (1999), Honicke and Broadbent (2016) and other scholars. It was also found that motivation, learning strategies, and self-efficacy moderated the relationship between the quality of the e-learning platform, the quality of the instructor’s teaching, and the learning environment and satisfaction with the e-learning outcomes, which is consistent with the findings of Kuo et al. (2014), Bandura (1977), and Borkotoky and Borah (2021). Therefore, lecturers or learners can increase internal and external incentives to enhance the perceived value of e-learning during the process of e-learning (Yahiaoui et al., 2022). It is recommended that lecturers in online courses give comprehensive evaluations based on the number of interactive exchanges on the platform, students’ enthusiasm in answering questions, the status of group discussions, and appropriate rewards based on the evaluations to enhance students’ satisfaction and pride. Secondly, it should pay close attention to the learning needs of students, such as the needs of college students to find a job, the demand for graduate school, the demand for bonuses, etc., which can be adjusted according to the different needs of the students of the network courses, to stimulate the enthusiasm of the students of network learning (Zhang et al., 2021).

#### 4.1.2. External factors affecting the effectiveness of college students’ online learning

Since 2020, the rapid development of e-learning platforms such as Tencent conference, MOOC, Rain Classroom, and Nail has provided students with a good learning platform. However, the quality of these platforms varies, and improvement is needed (Yuanyuan, 2022). This study has identified external factors that can directly or indirectly influence college students’ satisfaction with e-learning. These factors include the quality of the e-learning platform, the e-learning environment, and teacher support. Among these factors, the quality of the e-learning platform and teacher support are the most significant (Herrador-Alcaide et al., 2020). Therefore, long-lasting e-learning platforms should promptly adapt to societal and students’ learning needs by implementing technological changes and updating shared learning resources. Additionally, they should always maintain the innovative and social nature of e-learning resources. Furthermore, in terms of teacher support, interviews with students revealed that the teacher’s delivery style, choice of materials, teaching strategies, and proficiency in instructional tools significantly impact students’ satisfaction with learning outcomes, which supports the findings of Al Mamun et al. (2020) and Fraser et al. (1986). Hence, lecturers should strengthen interactive communication with students (Zhang et al., 2017), utilize pop-up functions and online Q&A during online lectures to encourage student participation and cultivate their interest. Moreover, teachers should adjust their teaching methods to enhance student engagement and optimize lesson design (Wang et al., 2021).

Scholars such as Allen and Fraser (2007) have identified the learning environment as an important factor influencing the effectiveness of online learning. In terms of family environment, due to the COVID-19 epidemic, college students are currently constrained to their homes, making the family environment the primary setting for their learning (Nainggolan, 2021). It is crucial to create a quiet and comfortable learning environment within the family. Furthermore, parents and teachers should communicate promptly with students and enhance supervision to improve the effectiveness of learning (Playle and Mullarkey, 1998). When online learning is implemented in schools, it is vital to promptly establish improved corresponding policies, enhance supporting measures such as software and hardware suitable for online learning, and closely monitor the psychological and physical changes of students (Zhai et al., 2020). These actions will contribute to the creation of a favorable learning environment.

### 4.2. LICE model combined with S-O-R model to explain the effects of factors on satisfaction with online learning effectiveness

This study constructs the LIPE model, which combines the LICE model with the S-O-R model, to explore how students’ satisfaction with learning can be improved from various perspectives. In the LICE theoretical model, the individual factor is considered the key factor, the instructor factor is an important factor, and the learning environment acts as a safeguard. Additionally, the learning platform serves as a supporting factor, and these various influences interact with each other (Lin et al., 2020). The inclusion of the S-O-R model in this integrated model provides a more scientifically rigorous explanation of the relationship between these influences. It elucidates the changes in students’ psychological cognition during the learning process, as well as their subsequent learning intentions and behavioral responses. Moreover, it better explains how adjustments and refinements of external influences, along with the internal factors of college students, contribute to the enhancement of learning satisfaction in an environment that receives external stimuli. This finding is consistent with the conclusions drawn by Guihua Zhang and other scholars (Zhang et al., 2021).

The results of the study showed that the changes in the learning environment, learning styles, learning methods, teaching methods, and other factors, as well as the different learning motives, learning strategies, and learning abilities of college students themselves, have led to changes in satisfaction with the effectiveness of e-learning.

### 4.3. E-learning applications in education

**Figure 7:**
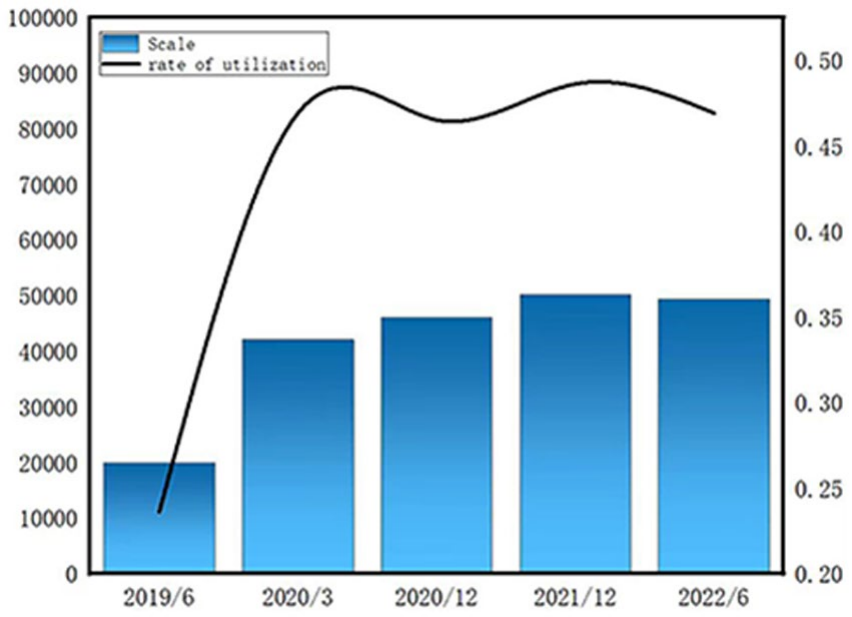
Scale and usage of online education from 2020 to 2022. Data from China’s 48th, 49th and 50th statistical report on the development of the Internet in China.

In December 2022, Chinese provinces gradually abolished the nucleic acid standing inspection, and the social side gradually opened up the epidemic control, while countries such as the United States had already begun to liberalize the restrictions on the prevention and control of epidemics long before that. The COVID-19 pandemic is over, but public health emergencies and damage caused by unforeseen factors are unpredictable. Therefore, the end of the pandemic has translated these lessons into clear guidelines and practices so that preventive and corrective actions can be taken promptly when exceptional circumstances arise. Figure 7 shows a sharp increase in the number of online learners in the network from 2019 until 2022. Network learning has become a college student learning life and should not be taken as a lesser method of learning. With students returning to offline learning, the question arises about the future of online learning and whether it should be abandoned. However, more than three-quarters of teachers preferred online instruction during the pandemic, and in the post-pandemic era, most prefer physical and blended instruction (Saha et al., 2022). Therefore, the development of online learning needs to change with the times. It is necessary to combine traditional learning methods with online learning to create a comprehensive approach (Edem Adzovie and Jibril, 2022).

## 5. Conclusion

The main purpose of this study was to investigate the factors that influence satisfaction with online learning outcomes. It primarily used structural equation modeling to analyze the effects of various factors on e-learning satisfaction. Specifically, the study examined the quality of e-learning platforms, the quality of teachers’ teaching, the e-learning environment, individual motivation in e-learning, learning strategies, and the effectiveness of self-directed learning. According to previous research, the quality of e-learning platforms, the quality of teachers’ teaching, the e-learning environment, and personal factors are all key determinants of satisfaction with e-learning outcomes. The study also revealed that college students’ e-learning motivation, learning strategies, and self-directed learning efficacy serve as mediators in their evaluation of e-learning satisfaction.

This study further verifies the deepening and presents the LIPE model, which was created based on the LICE model developed by Zhang Jiahua scholars. The LIPE model demonstrates that the learner’s factors, such as e-learning motivation, learning strategies, and self-learning efficacy, contribute to the internal factors that enhance the effectiveness of network learning. On the other hand, the quality of the network learning platform, the quality of the teacher’s lectures, and the network learning environment are identified as external factors that affect network learning effectiveness. The study reveals that a higher quality e-learning platform, better teacher instruction, and improved learning environment result in better e-learning outcomes. Additionally, the study highlights that e-learning motivation, learning strategies, and self-learning efficacy have a significant positive impact on learning effectiveness. These findings provide valuable insights for students themselves, as well as for improving school policies, enhancing the learning environment, developing online teaching platforms, refining teaching methods, and enhancing teaching quality.

According to research, e-learning, although it was imposed as a solution in a particular era, has always existed as a convenient way of learning and will continue to exist beyond this era. Therefore we should maximize the benefits of each learning method and keep optimizing it to minimize the drawbacks of the learning process.

### 5.1. Limitations and future directions

When evaluating the current study, several limitations need to be addressed. Firstly, the study only investigated undergraduate students, excluding master’s degree, college, and junior and senior high school students. This limitation affects the perspective and scope of the study. In future research, it would be beneficial to include additional levels of scope to provide a more comprehensive understanding of the topic. Another limitation of the study is that it is a longitudinal study spanning two periods during and at the end of the COVID-19 epidemic. To ensure a more accurate examination of the e-learning status of college students, it is recommended to extend the observation period to continuously track their experiences over a longer period. This would allow for a more robust understanding of changes in satisfaction with e-learning effects. Furthermore, the study primarily focuses on four aspects: the quality of the e-learning platform, the quality of the teacher’s instruction, the e-learning environment, and personal factors. However, it is important to note that satisfaction with e-learning effects is influenced by various factors. Therefore, future studies should consider incorporating more refined and comprehensive factors to provide a more nuanced analysis.

## Data availability statement

The raw data supporting the conclusions of this article will be made available by the authors, without undue reservation.

## Ethics statement

Ethical review and approval were not required for the study on human participants by the local legislation and institutional requirements. The patients/participants provided their written informed consent to participate in this study. Written informed consent was obtained from the individual (s) for the publication of any potentially identifiable images or data included in this article.

## Author contributions

XD, RRW, and XFH participated in the design, investigation, data analysis, and writing of the manuscript. XXW, YTH, YL, YW, and CYG participated in data collection and analysis. RQK participated in the investigation and evaluation of the study. YZ and BG participated in the design, investigation, and evaluation of the study and contributed to critical revision. All authors contributed to the article and approved the submitted version.
